# Functional connectivity correlates of sequence memory decline in healthy older adults

**DOI:** 10.1007/s11357-026-02125-x

**Published:** 2026-01-30

**Authors:** Catalina Trujillo-Llano, Nina M. Ehrhardt, Anna Elisabeth Fromm, Friederike Thams, Dayana Hayek, Shu-Chen Li, Agnes Flöel, Daria Antonenko

**Affiliations:** 1 Department of Neurology, Universitätsmedizin Greifswald, Greifswald, Germany; 2 Chair of Lifespan Developmental Neuroscience, Technische Universität Dresden, Dresden, Germany; 3 Centre for Tactile Internet With Human-in-the-Loop, Technische Universität Dresden, Dresden, Germany; 4 German Centre for Neurodegenerative Diseases (DZNE), Site Rostock/Greifswald, Germany

**Keywords:** Serial order memory, Episodic memory, Aging, Resting-state functional connectivity, Hippocampus, Default mode network

## Abstract

Episodic sequence memory is crucial for daily functioning and typically declines during aging. However, the neural mechanisms underlying this decline remain poorly understood. We examined the resting-state functional connectivity (FC) correlates of sequence memory in healthy older adults (OA), with a young adult (YA) group included for comparison. Thirty-eight OA (mean ± SD age: 69.9 ± 3.9 years; 24 women) and 20 YA (mean ± SD age: 24.2 ± 3.4 years; 14 women) completed a sequence memory task and resting-state functional magnetic resonance imaging (rsfMRI). Participants encoded sequences of greyscale pictures presented in colored frames and retrieved their sequential order. We compared the sequence memory performance (% correct) between age groups and examined associations between performance and seed-based FC patterns within each group, focusing on hippocampal and default mode network (DMN) regions. OA exhibited poorer sequence memory performance than YA. In OA, lower performance was associated with reduced FC between the left posterior hippocampus and DMN hubs, and the DMN and right posterior parietal cortex, suggesting that reduced intrinsic coupling within memory networks and between memory and attention networks contributes to sequence memory decline in aging. In YA, superior sequence memory performance correlated with higher FC between the left posterior hippocampus and right medial occipital cortex, indicating that hippocampal-perceptual integration benefits memory function. The FC in these networks did not differ between age groups, indicating a potential shift in memory-relevant connectivity in older adults rather than reduced network function per se. Our findings provide novel evidence of large-scale network correlates underlying sequence memory decline in old age and suggest qualitatively distinct connectivity patterns supporting sequence memory in young and older adults.

**Supplementary information:**

The online version contains supplementary material available at https://doi.org/10.1007/s11357-026-02125-x.

## Introduction

Sequence memory, the ability to recall the order of events, is a core component of episodic memory essential for daily functioning [1, 2]. It enables the prediction of future events and planning goal-directed actions [1]. Age-related decline in sequence memory [3, 4] can emerge as early as middle age [5] and may precede broader cognitive deterioration [6–8], reflecting generalized difficulties in integrating contextual features into memory [9]. Given its sensitivity to aging and relevance to everyday functioning [10], sequence memory may serve as a potential early marker to distinguish pathological from healthy aging [11, 12]. Despite extensive behavioral research, few studies have explored the neural underpinnings of sequence memory in older adults (OA [13–15]).

Sequence memory relies on the binding of items with their temporal context [16]. This process is supported by the hippocampus in coordination with a distributed cortical network encompassing posterior parietal and medial prefrontal cortices [1, 17], which together constitute the medial temporal subsystem of the default mode network (DMN [18, 19]). Task-based fMRI studies in young adults (YA) demonstrate hippocampal and DMN involvement in sequence encoding and retrieval [20–25]. The functional connectivity among these regions is critical for sequential memory [26, 27]. In contrast, OA show activity changes in these regions during temporal order retrieval compared to YA, including failure to suppress medial prefrontal areas, reduced posterior parietal activity, and increased medial temporal activity linked to poorer retrieval accuracy [13–15]. OA also exhibit reduced hippocampal and posterior medial activity at event boundaries, which help structure continuous experiences into temporally ordered segments [28].

While these task-based fMRI studies allow to delineate activity patterns and their age-related changes in sequence memory, resting-state functional connectivity (rsFC) may offer complementary insights into the mechanisms underlying its decline in aging. rsFC measures the temporal correlations in spontaneous low-frequency fluctuations between brain regions in the absence of task demands, capturing the intrinsic functional architecture of large-scale networks [29]. Examining intrinsic hippocampal-DMN coupling is particularly relevant because sequence memory relies on the coordinated interaction of these distributed systems [26, 27], and baseline network organization may index the system's capacity for this coordination. Additionally, given that hippocampal-DMN networks are particularly vulnerable to aging [30], disruptions in their intrinsic coupling may impair the integration and retrieval of sequential information. Indeed, reduced DMN rsFC has been associated with episodic memory impairments in OA [31, 32], likely reflecting compromised network integrity [33]. Thus, rsFC may reveal intrinsic network-level mechanisms that contribute to sequence memory decline in aging. However, the relationship between hippocampal-DMN rsFC patterns and sequence memory performance in OA remains unexplored.

To address this gap, we examined the rsFC correlates of sequence memory in healthy OA. A sex-matched group of YA was included to provide a functional reference of performance typical in young adulthood. We first compared the behavioral performance between age groups. Then, we examined the associations between sequence memory performance and seed-based rsFC within each age group, focusing on anterior and posterior hippocampus and DMN regions. Finally, we explored whether the rsFC within the performance-relevant networks differed between age groups. In line with previous research, we hypothesized that OA would demonstrate lower sequence memory performance compared to YA. We further hypothesized that, in OA, poorer performance would be associated with lower rsFC between hippocampal and DMN regions, reflecting age-related vulnerability of memory networks and diminished hippocampal-cortical integration [31, 32]. While our primary focus was on OA, we also examined rsFC-performance associations in YA in an exploratory manner. Characterizing the rsFC patterns associated with sequence memory performance within each group allowed us to assess whether sequence memory in aging is supported by qualitatively distinct networks, consistent with an age-related shift in the neural architecture underlying sequence memory. Furthermore, by comparing the rsFC strength within the performance-relevant networks identified in each group, we evaluated whether age-related differences in sequence memory are accompanied by reductions in intrinsic connectivity.

## Methods

### Participants

The study included 58 healthy participants, comprising 38 OA (24 women; mean ± SD age, 69.9 ± 3.9 years, range: 63–79 years) and 20 YA serving as a control group (14 women; mean ± SD age, 24.2 ± 3.4 years, range: 20–33 years). All participants were right-handed, native German speakers with normal or corrected-to-normal hearing and vision. None reported a history of alcohol/drug abuse, psychiatric or neurological disease or MRI contraindications. OA participants had preserved cognitive function, scoring above −1.5 standard deviations (*z*-scores) on age- and education-adjusted German norms of the CERAD Neuropsychological Assessment Battery (https://www.memoryclinic.ch).

The groups were matched for sex but marginally differed in years of education (Table 1). Therefore, education was included as a covariate in the between-group behavioral analysis (see the Data Analysis section). Demographic and general cognitive information are detailed in Table 1. All participants provided informed consent in accordance with the Declaration of Helsinki and received compensation for their participation. The Ethics Committee of the University Medicine Greifswald approved the study.

**Table 1:** Demographic, general cognitive, and sequence memory data

	OA (***n*** = 38)	YA (***n*** = 20)	OA vs. YA Statistics
Demographics			
Age (years)	69.97 (3.90)	24.20 (3.37)	*t*_(56)_ = 44.45; *p* < 0.001^*** a^; *d* = 12.28
Education (years)	16.45 (2.78)	17.75 (1.61)	*t*_(56)_ = − 1.93; *p* = 0.059 ^a^; *d* = −0.53
Sex (F: M)	24: 14	14: 6	*χ*^2^_(1)_ = 0.27; *p* = 0.602 ^b^
General cognitive state			
CERAD total *z*-score	−0.02 (0.38)	−	−
Sequence memory			
Total retrieval score (%)	64.0 (9.06)	79.45 (9.83)	*F*_(1,55)_ = 30.13; *p* < 0.001^***c^; *ηp*^*2*^ = 0.35

Data are presented as mean (*SD*), except for sex.

Significance coding: ^*^p < 0.05; ^**^p < 0.01; ^***^p < 0.001.

^a^ *p*-value calculated via independent *t*-test (*t*).

^b^ *p*-value calculated via chi-squared test (*χ*^2^).

^c^ *p*-value calculated via ANCOVA with education as covariate.

CERAD: Consortium to Establish a Registry for Alzheimer's Disease Neuropsychological Assessment Battery; OA: Older adults; YA: Young adults.

### Materials

#### Sequence memory task

Participants completed a computerized sequence memory task adapted from previous studies [34, 35]. Stimuli consisted of greyscale images of non-human objects from the MIT object database (http://cvcl.mit.edu/MM/uniqueObjects.html), presented sequentially within colored frames. The images contained no negative valence content (i.e., weapons, spiders) or text. Frame colors were selected for maximum visual distinctiveness.

The task comprised two phases: encoding and retrieval. During encoding, participants viewed the greyscale images presented sequentially within colored frames and were instructed to apply two encoding strategies to each image in all blocks: (a) a color-matching strategy, where they imagined each object in its color frame and indicated whether the color suited the object via button press, and (b) an object-interaction strategy, where they imagined each object interacting with the previously presented one. The encoding phase was divided into 10 blocks, each consisting of five lists of five images (totaling 25 images). Frame colors changed between lists. Image presentation was fixed at 3500 ms, followed by a 500 ms fixation cross and 2000 ms inter-trial interval.

The retrieval phase immediately followed each encoding block. During retrieval, participants viewed pairs of objects from the preceding encoding block, presented side by side in grey frames. Each pair comprised either the first and fourth or the second and fifth objects from each list. The left-to-right arrangement of image pairs had a 50% chance of matching their original sequential order, with the order pseudorandomized across trials. Participants indicated whether the order of the objects matched the order of the encoded sequence and rated their confidence using a four-option forced-choice scale. Each retrieval block included 10 trials.

The complete task consisted of 10 blocks, resulting in 100 retrieval trials. Sequence memory performance was measured as the percentage of correct responses across all trials. Before testing, participants completed a training session of four retrieval trials to ensure comprehension of task instructions.

#### Neuroimaging acquisition

Data acquisition and processing followed the guidelines of the Organization for Human Brain Mapping [36, 37]. MRI data were acquired in a separate session from the behavioral memory task. The interval between sessions varied across participants (mean ± SD = 18.5 ± 47.9 days, range: −95.8–132.77 days) and no resting-state scan was conducted immediately after the behavioral task. Thus, our rsFC results most likely reflect intrinsic connectivity rather than transient post-task effects. Data were acquired on a 3 T Siemens Verio scanner equipped with a 32-channel head coil. High-resolution T1-weighted images were acquired using a magnetization-prepared rapid gradient echo (MPRAGE) sequence with the following parameters: voxel size = 1 mm^3^ isotropic; TR = 1690 ms; TE = 2.52 ms; inversion time = 900 ms; flip angle = 9°; matrix size = 256 × 256 mm; number of slices = 176. Resting-state fMRI scans were obtained using an echo-planar imaging (EPI) sequence with the following parameters: voxel size = 3 mm^3^ isotropic; TR = 2000 ms; TE = 30 ms; flip angle = 90°; matrix size = 384 × 384 mm; number of slices = 34; descending acquisition; number of volumes = 176; acquisition time = 6 min. For this sequence, participants were instructed to keep their eyes closed, avoid moving or falling asleep, and not to think about anything in particular.

### Data analysis

#### Behavioral data analysis

Demographic data were compared between groups using independent t-tests and Chi-squared tests (*χ*^*2*^), as appropriate. Sequence memory performance was compared between groups using ANCOVA, including education as a covariate. A complementary analysis including both education and sex as covariates was conducted to verify the robustness of the group effect. All statistical tests were conducted with a two-sided alpha level of 0.05. Effect sizes were calculated through partial eta-squared (*ηp*^*2*^). Analyses were performed using R Statistical Software (v 4.4.0; [38]).

#### Resting-state functional connectivity preprocessing and analysis

Functional and anatomical data were preprocessed and analyzed using the CONN toolbox v22a [39, 40] and SPM12 [41] running on MATLAB 2023b.

##### Preprocessing pipeline

Data were preprocessed using a flexible preprocessing pipeline [42] consisting of the following steps: (1) Realignment: Functional data were realigned using the SPM realign & unwarp procedure [43]. All scans were coregistered to a reference image (first scan of the first session) using a least squares approach and a 6-parameter rigid body transformation [44] and resampled using b-spline interpolation to correct for motion and magnetic susceptibility interactions. (2) Slice-timing correction: Temporal misalignment between different slices of the functional data (acquired in descending order) was corrected following the SPM slice-timing correction procedure [45, 46], using sinc temporal interpolation to resample each slice BOLD time-series to a common mid-acquisition time. (3) Outlier detection: Potential outlier scans were identified using Artifact Detection Tools (ART [47]) as acquisitions with framewise displacement above 0.9 mm or global BOLD signal changes above five standard deviations [48, 49]. A reference BOLD image was computed for each subject by averaging all scans excluding outliers. (4) Normalization and segmentation: Functional and anatomical data were normalized into standard MNI space, segmented into grey matter, white matter, and CSF tissue classes, and resampled to 2 mm isotropic voxels following a direct normalization procedure [48, 50]. This was implemented using SPM's unified segmentation and normalization algorithm [51, 52] with the default IXI-549 tissue probability map template. This template is derived from a large adult sample (*n* = 549) spanning a wide age range (mean ± SD = 48.6 ± 16.6, range: 20–86 years), making it suitable for our mixed YA and OA cohort. Visual inspection confirmed appropriate normalization across participants. (5) Spatial smoothing: Functional data were smoothed using spatial convolution with a Gaussian kernel of 8 mm full-width half maximum (FWHM).

##### Denoising

Preprocessed functional data were denoised using a standard denoising pipeline [42] to remove physiological and non-neural sources of variance. The following confounding factors were regressed from the BOLD signal: (a) white matter time series (5 CompCor noise components), (b) CSF time series (5 CompCor noise components), (c) motion parameters and their first-order derivatives (12 factors) [53], (d) outlier scans (below 15 factors) [49], (e) session effects and their first-order derivatives (2 factors), and (f) linear trends within each functional run (2 factors). CompCor [54, 55] noise components within white matter and CSF were estimated by computing the average BOLD signal and the largest principal components orthogonal to the BOLD average, motion parameters, and outlier scans within each subject's eroded segmentation masks. Following regression, high-pass filtering at 0.01 Hz was applied to the BOLD time series [56] to remove low-frequency drift. Based on the number of noise terms included in this denoising strategy, the effective degrees of freedom of the BOLD signal after denoising were estimated to range from 129.6 to 144 (mean = 143) across all subjects [48]. Finally, all preprocessing and denoising outputs were visually inspected and considered appropriate for analysis.

We conducted seed-based connectivity analysis to measure rsFC patterns of the hippocampus and DMN. First, we generated seed-to-voxel connectivity maps of three regions of interest (ROIs): the bilateral anterior hippocampus (aHPC), the bilateral posterior hippocampus (pHPC), and the posterior cingulate cortex (PCC). For the hippocampal seeds, we placed 5-mm bilateral spherical ROIs at well-established meta-analytic MNI coordinates [57]: right aHPC (*x* = 28, *y* = −16, *z* = −18), left aHPC (*x* = −26, *y* = −16, *z* = −18), right pHPC (*x* = 28, *y* = −30, *z* = −8), and left pHPC (*x* = −26, *y* = −30, *z* = −8). This anterior–posterior hippocampal division was based on differential involvement and interactions with the DMN in episodic memory processes [58, 59], age-related rsFC patterns [60], and stimulus modality preferences [61]. For DMN connectivity, we used a PCC ROI (MNI coordinates: *x* = 1, *y* = −61, *z* = 38; volume: 38.66 mm^3^) from the CONN toolbox. This ROI was derived through independent component analysis of the Human Connectome Project dataset, which included 497 participants [40]. Functional connectivity strength was quantified using Fisher-transformed bivariate correlation coefficients between the averaged time course of the seed region and the time course of each voxel throughout the whole brain.

Next, we examined associations between sequence memory performance and rsFC within each group separately. Linear regression analyses were conducted using the seed-specific connectivity maps as the dependent variable and sequence memory performance as the regressor of interest. Complementary analyses were conducted including age, sex, and head motion (average framewise displacement across non-outlier scans [62]) as covariates. Due to the modest sample size and exploratory nature of our study, statistical thresholds were set at a voxel-level threshold of *p*-uncorrected < 0.005 and a cluster-level threshold of *p*-FDR corrected < 0.05. This approach aligns with previous rsFC studies using similar analytical frameworks in aging populations [63–65]. Although more conservative voxel-level thresholds (i.e., *p* < 0.001) have been recommended to minimize false positives [66], these can substantially increase Type II errors, particularly in studies with modest statistical power [67]. Other simulation studies suggest that using slightly more liberal voxel-level thresholds (i.e., *p* < 0.005) combined with appropriate cluster-extent criteria (i.e., ≥ 10 contiguous voxels) can adequately balance Type I and Type II error rates [67]. All statistical tests were parametric and two-tailed. Significant results were plotted using BrainNet Viewer v.1.63 [68] and anatomically labeled using the Harvard–Oxford Atlas [69].

Finally, we explored whether the rsFC within performance-relevant networks differed between age groups. We extracted the individual connectivity values from the networks showing significant associations with sequence memory performance in the within-group analyses (i.e., left pHPC-DMN and DMN-posterior parietal cortex for OA, and left pHPC-intracalcarine cortex for YA) for all participants. Group differences in rsFC of each network were tested using separate linear regression models with age group as predictor in R Statistical Software (v 4.4.0; [38]). Complementary analyses were conducted including sex and head motion as covariates. We report unstandardized beta coefficients, *p*-values, and 95% confidence intervals (CIs) to characterize the precision of these estimates. Effect sizes were calculated through partial eta-squared (*ηp*^*2*^). Finally, to evaluate statistical power for between-group comparisons, we conducted a sensitivity analysis using G*Power v. 3.1 (α = 0.05, two-tailed; power = 0.80). This analysis indicated that our sample size provided adequate power to detect large effects (Cohen's *d* ≥ 0.79) […]".

## Results

### Behavioral results

OA exhibited lower sequence memory performance than YA [*F*_(1,55)_ = 30.13, *p* < 0.001, *η*_*p*_^*2*^ = 0.35] – see Table 1. This effect persisted when including education and sex as covariates [*F*_(1,54)_ = 27.98, *p* < 0.001, *ηp*^*2*^ = 0.34].

### Associations between resting-state functional connectivity and sequence memory performance

**Fig. 1:**
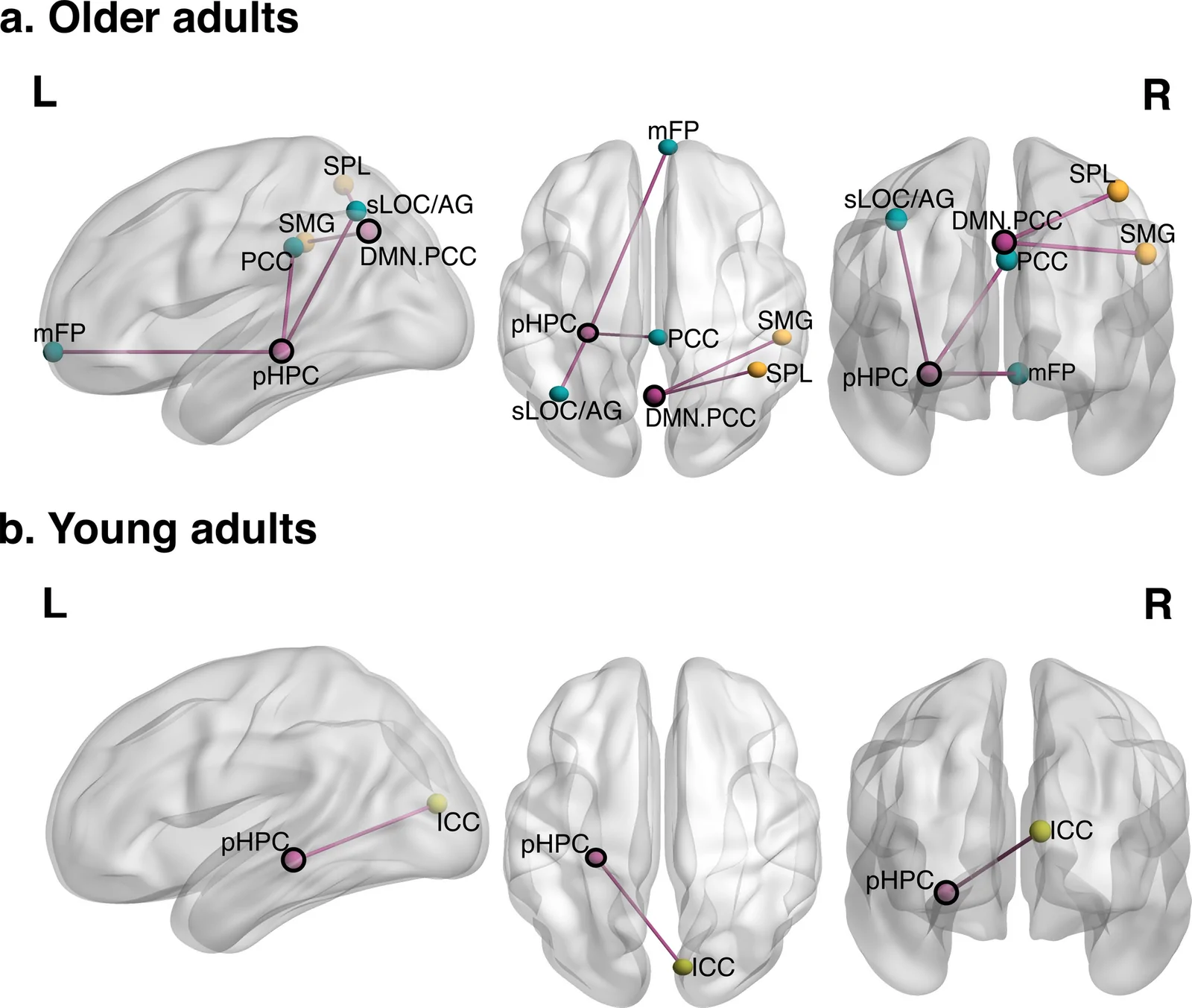
Resting-state functional connectivity networks associated with sequence memory performance. Brain maps display significant correlations between sequence memory performance and seed-based resting-state functional connectivity in **(a)** older adults and **(b)** young adults. Seed regions are shown as pink spheres outlined in black. Pink edges connect each seed to its associated significant clusters. Colored nodes indicate significant clusters associated with each seed at peak MNI coordinates. **(a)** In older adults, lower performance was associated with reduced connectivity between the left pHPC and DMN regions (cyan nodes: mFP, sLOC/AG, and PCC) and between the DMN.PCC and posterior parietal regions (yellow nodes: SMG and SPL). **(b)** In young adults, higher performance was associated with increased connectivity between the left pHPC and right intracalcarine cortex (green node: ICC). Statistical threshold: voxel-level *p*-uncorrected < 0.005; cluster-level *p*-FDR corrected < 0.05. Results are presented in MNI space and neurological convention. Seeds: *pHPC* posterior hippocampus, *DMN.PCC*  posterior cingulate cortex (default mode network hub), Significant associated clusters: *ICC* intracalcarine cortex, *mFP*  medial frontal pole, *sLOC/AG*  superior lateral occipital cortex/angular gyrus, *SMG*  supramarginal gyrus, *SPL*  superior parietal lobule, *PCC*  posterior cingulate cortex, Hemisphere labels: *L*  left, *R*  right

In OA, lower sequence memory performance was associated with lower rsFC between the left pHPC and core DMN hubs, including left lateral occipito-parietal (peak voxel MNI coordinates: *x* = −38, *y* = −60, *z* = 46; *T*_36_ = 4.31; *k* = 237), posterior cingulate (peak voxel MNI coordinates: *x* = 2, *y* = −32, *z* = 32; *T*_36_ = 4.55; *k* = 208), and medial prefrontal areas (peak voxel MNI coordinates: *x* = 6, *y* = 62, *z* = −8; *T*_36_ = 4.24; *k* = 181) – see Fig. 1a and Supplementary Table S1. Additionally, lower sequence memory performance correlated with lower rsFC between the DMN and right posterior parietal areas, including the supramarginal gyrus (peak voxel MNI coordinates: *x* = 52, *y* = −32, *z* = 34; *T*_36_ = 4.75; *k* = 290) and superior parietal lobule (peak voxel MNI coordinates: *x* = 42, *y* = −48, *z* = 56; *T*_36_ = 4.94; *k* = 274) – see Fig. 1a and Supplementary Table S1. No significant associations emerged for the bilateral aHPC and right pHPC seeds.

In YA, higher sequence memory performance was associated with higher rsFC between the left pHPC and right medial occipital cortex, including the intracalcarine cortex (peak voxel MNI coordinates: *x* = 8, *y* = −82, *z* = 14; *T*_18_ = 4.44; *k* = 216) – see Fig. 1b and Supplementary Table S1. No significant associations emerged for the bilateral aHPC, right pHPC, and DMN seeds.

In both groups, these results were consistent when including age, sex, and head motion as covariates (see Supplementary Material 1 and Supplementary Table S2).

### Between-group comparisons of resting-state functional connectivity in performance-relevant networks

We found no evidence of group differences in rsFC in performance-relevant networks. No group differences were observed in the rsFC between the left pHPC and DMN clusters (*β* = 0.04, 95% CI [−0.03, 0.11], *p* = 0.253, *ηp*^*2*^ = 0.02), the DMN and posterior parietal cortex clusters (*β* = 0.04, 95% CI [−0.08, 0.16], *p* = 0.519, *ηp*^*2*^ = 0.01), or the left pHPC and medial occipital cortex cluster (*β* = 0.00, 95% CI [−0.06, 0.06], *p* = 0.988, *ηp*^*2*^ < 0.001) – see Supplementary Fig. S1.

These results remained consistent when including sex and head motion as covariates (left pHPC-DMN: *β* = 0.03, 95% CI [−0.05, 0.11], *p* = 0.467, *ηp*^*2*^ = 0.02; DMN-posterior parietal: *β* = 0.13, 95% CI [−0.01, 0.26], *p* = 0.069, *ηp*^*2*^ = 0.01; left pHPC-intracalcarine: *β* = 0.01, 95% CI [−0.05, 0.08], *p* = 0.710, *ηp*^*2*^ < 0.001).

## Discussion

This study examined the rsFC correlates of sequence memory in healthy OA and YA. OA showed lower sequence memory performance than YA, which was associated with lower rsFC between the left pHPC and key DMN hubs, as well as lower rsFC between the DMN and right posterior parietal cortex. These results suggest that reduced intrinsic coupling within episodic memory networks and between memory and attention-related networks contributes to sequence memory impairments in aging. In contrast, in YA, superior sequence memory performance was associated with higher rsFC between the left pHPC and right medial occipital cortex, indicating that hippocampal-perceptual integration supports optimal performance in YA. However, the rsFC strength per se in these networks did not differ between age groups. Together, our findings provide novel evidence of large-scale network mechanisms underlying sequence memory decline in aging and suggest qualitatively distinct connectivity patterns supporting sequence memory within each age group.

Consistent with prior studies [3–9], OA showed worse sequence memory performance than YA. Within the OA group, lower sequence memory performance correlated with decreased rsFC between the left pHPC and DMN hubs (left lateral occipito-parietal, posterior cingulate, and medial prefrontal cortices). These regions comprise the posterior medial network, which supports episodic reconstruction by integrating spatial, temporal, and contextual features into coherent event representations [70, 71]. Our findings suggest that lower intrinsic DMN connectivity, critical for episodic memory, contributes to sequence memory deficits in OA. These results align with the DMN integrity hypothesis [33], where diminished DMN connectivity is associated with episodic memory decline in aging [31, 32, 72] and in patients with mild cognitive impairment and Alzheimer’s disease [73]. Our findings extend this evidence by identifying reduced pHPC-DMN connectivity as a potential specific neural correlate of sequence memory deficits in aging. They also complement task-fMRI evidence showing activity changes in DMN nodes during temporal order retrieval in OA [13–15]. Future research should examine how these rsFC patterns correlate with task-evoked activity during sequence memory performance in OA.

Additionally, poorer sequence memory performance was associated with lower rsFC between the DMN and right posterior parietal cortex (superior parietal lobule and supramarginal gyrus). While the DMN supports internally driven retrieval processes [59], posterior parietal regions contribute to allocating attentional resources during memory retrieval [74]. Reduced DMN-parietal coupling may reflect decreased integration between memory and attention systems, potentially affecting the ability to maintain and manipulate temporal order information during retrieval. Converging evidence has shown positive associations between DMN-posterior parietal rsFC and overall episodic memory performance in aging and early Alzheimer’s disease [75, 76]. We extend this evidence by showing that reduced intrinsic DMN-posterior parietal coupling is a potential mechanism specifically underlying sequence memory decline in aging. Together, our findings suggest that lower hippocampal-DMN and DMN-posterior parietal rsFC contribute to poorer sequence memory performance in aging.

In contrast, in YA, superior sequence memory performance was associated with higher rsFC between the pHPC and medial primary visual cortex (intracalcarine cortex), suggesting that hippocampal-perceptual integration supports optimal performance. The hippocampus supports item-temporal context binding [77], while the primary visual cortex processes low-level features and may reactivate perceptual representations during retrieval [78]. In our task, the use of visual imagery and relational encoding strategies likely enhanced the perceptual richness of object sequences and the engagement of early visual areas. Stronger pHPC-visual cortex FC may facilitate access to these perceptual representations during retrieval, consistent with models proposing that episodic memory involves hippocampal interactions with sensory cortices to reactivate modality-specific features originally engaged during encoding [79, 80]. Our findings extend previous task-fMRI evidence demonstrating hippocampal [20, 22, 25] and medial occipital engagement during temporal order memory tasks in YA [13] by showing that intrinsic connectivity between these regions supports successful sequence memory performance. Future studies should examine how these rsFC patterns are associated with task-evoked activity during sequential memory tasks in YA.

The absence of this hippocampal-occipital rsFC pattern in OA may reflect several age-related changes. First, age-related decline in visual processing efficiency [81] and visual cortex function [82, 83] may reduce the precision and fidelity of perceptual representations [84] that could be reactivated during retrieval, limiting the utility of hippocampal-visual cortex coupling for memory performance. Second, OA may rely more on gist-based rather than perceptually detailed representations during memory encoding and retrieval, which may engage more distributed, higher-order networks rather than specific hippocampal-sensory networks [85]. Consistent with this interpretation, OA in our study showed performance-related rsFC patterns involving distributed memory (pHPC-DMN) and memory-attention (DMN-parietal) networks rather than focal hippocampal-sensory networks. These qualitatively distinct rsFC patterns suggest that sequence memory in aging may rely on alternative neural mechanisms involving broader, higher-order rather than specific perceptual networks.

Although qualitatively distinct networks were associated with sequence memory performance within each age group, we found no evidence of group differences in the connectivity strength of these networks. Specifically, the rsFC between left pHPC and DMN hubs, DMN and posterior parietal cortex, and left pHPC and medial occipital cortex did not show evidence of age-group differences. While age-related reductions in rsFC are frequently reported, such effects can be subtle, variable, and influenced by sample characteristics and methodological approaches [30]. Our findings suggest that these networks are preserved across age but distinctively support sequence memory within each group, with OA relying on distributed memory and attentional networks, while YA benefit from more specific memory-perceptual integration mechanisms. Notably, these associations emerged specifically from the pHPC in both groups, consistent with its central role in memory retrieval [58, 59] and processing of pictorial stimuli [61], characteristics that align with our task demands.

Our findings have several mechanistic and clinical implications. Mechanistically, they provide novel evidence of rsFC correlates underlying sequence memory decline in OA, extending findings from previous behavioral [3–9] and task-fMRI studies [13–15]. Clinically, given that age-related memory disorders such as mild cognitive impairment and Alzheimer’s disease involve sequence memory deficits [11, 12] and disruptions in similar networks identified here [30, 86], our findings underscore the relevance of further exploring sequence memory mechanisms in pathological aging. Furthermore, our findings may inform the preliminary selection of candidate target regions for future non-invasive brain stimulation (NIBS) studies aimed at supporting memory function in OA. For instance, the posterior parietal and hippocampal network nodes associated with lower sequence memory performance in our OA group could represent feasible targets for NIBS techniques such as transcranial direct current stimulation (tDCS), transcranial alternating current stimulation (tACS), or emerging approaches like transcranial temporal interference stimulation (tTIS). These techniques have shown promise in modulating episodic memory and large-scale networks across healthy and memory-impaired populations [63, 87, 88]. However, as our rsFC findings provide only correlational evidence, these potential targets require validation through task-based fMRI or electroencephalography (EEG), proof-of-concept neuromodulation (i.e., concurrent NIBS and neuroimaging), and multimodal studies.

### Limitations and future directions

Some limitations of our study should be considered. First, our YA sample size was relatively small. While comparable to previous sequence memory neuroimaging studies [13–15], this limits the generalizability and replicability of the within-group YA findings and the statistical power to detect between-group rsFC differences. Our study was sufficiently powered to detect large effects (Cohen's *d* ≥ 0.79), but the observed effect sizes in the between-group rsFC comparisons (*ηp*^*2*^ ≤ 0.02; equivalent to *d* ≤ 0.31) were small and fell substantially below this threshold, indicating considerable risk of Type II error. Therefore, our null findings should be interpreted cautiously, as modest between-group differences in rsFC may have remained undetected with the current sample size. Future studies with larger samples are essential to clarify age-related differences in rsFC in performance-relevant networks and the within-group YA associations identified here. Second, while our task effectively assessed sequence memory, it lacked a naturalistic context. Given that sequence memory relies on the integration of items and contextual features [16], which are richer in real-world scenarios, using more ecologically valid paradigms may provide deeper insights into sequence memory functions in daily life [28]. Third, our cross-sectional design and correlational analyses preclude causal interpretations. To strengthen mechanistic interpretations, future research should incorporate longitudinal designs to track individual trajectories of change in both connectivity patterns and memory performance over time. Furthermore, multimodal approaches, combining rsFC with task-based fMRI, structural connectivity, or high-temporal-resolution methods such as magnetoencephalography (MEG) and EEG, are crucial for more precisely characterizing the multi-level mechanisms underlying sequence memory decline in aging. Specifically, task-based fMRI examining connectivity before, during, and after memory task performance would enable direct comparison between intrinsic and task-evoked network dynamics. Additionally, dynamic functional connectivity analyses or perceptual processing tasks could further clarify hippocampal-visual contributions to memory with aging by revealing temporal dynamics and perceptual mechanisms that static rsFC cannot capture. Fourth, age-related atrophy, particularly in hippocampal and prefrontal regions, may introduce spatial misalignment during normalization and could influence voxel-wise localization and between-group rsFC comparisons. However, our normalization approach employed an age-diverse template (20–86 years) rather than a young-adult-only template, and total intracranial volume did not differ between groups, reducing the likelihood of major normalization differences. Moreover, previous studies in OA have reported similar group-level fMRI findings when using standard MNI and study-specific templates [89, 90]. Still, future studies may benefit from using age- or study-specific templates to further minimize potential misalignment. Finally, our study included only healthy OA, limiting generalizability to clinical populations such as mild cognitive impairment and Alzheimer's disease. However, characterizing the networks associated with sequence memory in healthy aging is essential for establishing baseline networks that should be targeted in pathological aging.

## Conclusion

Our findings indicate that sequence memory impairments in OA are associated with reduced rsFC between the pHPC and DMN, as well as between the DMN and posterior parietal cortex, suggesting weakened integration within episodic memory networks and between episodic and attention-related systems. In contrast, in YA higher sequence memory performance is associated with stronger hippocampal-visual cortex rsFC, indicating performance benefits from hippocampal-perceptual network integration. These findings reveal specific connectivity patterns that may serve as potential neural markers for the early detection of memory decline and targets for non-invasive brain stimulation interventions in aging populations.

## Supplementary information

Below is the link to the electronic supplementary material.

ESM 1 (ESM DOCX 365 KB)

## Data Availability

The data supporting this study's findings are available from the corresponding author upon reasonable request.
