# Supplementary material for: Functional connectivity correlates of sequence memory decline in healthy older adults
Source: GeroScience. 2026 Jan 30;48(6):8147–60. doi: 10.1007/s11357-026-02125-x (PMC13638877; doi:10.1007/s11357-026-02125-x)
Supplement: Supplementary file 1 [file 11357_2026_2125_MOESM1_ESM.docx]

**Functional connectivity correlates of sequence memory decline in healthy older adults**

Catalina Trujillo-Llano^1^, Nina M. Ehrhardt^1^, Anna Elisabeth Fromm^1^, Friederike Thams^1^, Dayana Hayek^1^, Shu-Chen Li^2,3^, Agnes Flöel^1,4^, Daria Antonenko^1*^

^1^ Department of Neurology, Universitätsmedizin Greifswald, Greifswald, Germany

^2^ Chair of Lifespan Developmental Neuroscience, Technische Universität Dresden, Dresden, Germany

^3^ Centre for Tactile Internet with Human-in-the-Loop, Technische Universität Dresden, Dresden, Germany

^4^ German Centre for Neurodegenerative Diseases (DZNE), Site Rostock/Greifswald, Germany

^*^Correspondence concerning this article should be addressed to Prof. Dr. Daria Antonenko

Universitätsmedizin Greifswald, Ferdinand-Sauerbruch-Straße, Greifswald, Germany, Zip code 17475. Phone: (+49) 3834866754. Email: daria.antonenko@med.uni-greifswald.de

Table of Contents

[1. Supplementary Material 1	2](#_Toc217058582)

[Associations between resting-state functional connectivity and sequence memory performance after covarying for age, sex, and head motion	2](#_Toc217058583)

[2. Supplementary tables	2](#_Toc217058584)

[Table S1. Resting-state functional connectivity patterns associated with sequence memory performance in older and young adults	2](#_Toc217058585)

[Table S2. Resting-state functional connectivity patterns associated with sequence memory performance in older and young adults after covarying for age, sex and head motion	3](#_Toc217058586)

[3. Supplementary figures	4](#_Toc217058587)

[Figure S1. Between-group comparisons of resting-state functional connectivity in performance-relevant networks.	4](#_Toc217058588)

# **1. Supplementary Material 1**

# **Associations between resting-state functional connectivity and sequence memory performance after covarying for age, sex, and head motion**

In OA, lower sequence memory performance was associated with lower rsFC between the left pHPC and key DMN hubs, including left lateral occipito-parietal (peak voxel MNI coordinates: *x* = -40, *y* = -60, *z* = 48; *T*_33_ = 4.80; *k* = 373), posterior cingulate (peak voxel MNI coordinates: *x* = -16, *y* = -44, *z* = 44; *T*_33_ = 5.36; *k* = 359), and medial prefrontal areas (peak voxel MNI coordinates: *x* = 6, *y* = 62, *z* = -8; *T*_33_ = 4.28; *k* = 189) – see Supplementary Table S2. Moreover, lower sequence memory performance correlated with lower rsFC between the DMN and right posterior parietal areas, including the supramarginal gyrus and parietal operculum cortex (peak voxel MNI coordinates: *x* = 52, *y* = -30, *z* = 34; *T*_33_ = 4.77; *k* = 303) – see Supplementary Table S2. No significant associations emerged for the bilateral aHPC and right pHPC seeds.

In YA, higher sequence memory performance was associated with higher rsFC between the left pHPC and right medial occipital cortex (peak voxel MNI coordinates: *x* = 10, *y* = -78, *z* = 16; *T*_15_ = 5.10; *k* = 296) – see Supplementary Table S2. No significant associations emerged for the bilateral aHPC, right pHPC, and DMN seeds.

# **2. Supplementary tables**

# **Table S1. Resting-state functional connectivity patterns associated with sequence memory performance in older and young adults**

| Group | Seed | Cluster size ^a^ | Brain region ^b^ | Peak statistics (T) ^c^ | Peak MNI Coordinates | | |
| --- | --- | --- | --- | --- | --- | --- | --- |
|  |  |  |  |  | ***x*** | ***y*** | ***z*** |
| OA | Left pHPC | 237 | Lateral occipital cortex, superior division_L | 4.7 | -36 | -66 | 46 |
|  |  |  | Angular gyrus_L | 4.1 | -40 | -58 | 44 |
|  |  |  | Angular gyrus_L | 3.2 | -40 | -56 | 44 |
|  |  | 208 | Cingulate gyrus, posterior division | 4.6 | 2 | -32 | 34 |
|  |  |  | Cingulate gyrus, posterior division | 4.3 | 10 | -30 | 34 |
|  |  | 181 | Frontal pole_R | 4.3 | 6 | 60 | -8 |
|  |  |  | Frontal pole_R | 3.9 | 10 | 58 | -8 |
|  |  |  | Frontal pole_L | 3.7 | -4 | 58 | -6 |
|  |  |  | Frontal medial cortex | 3.7 | 0 | 54 | -8 |
|  |  |  | Paracingulate gyrus_R | 3.7 | 6 | 52 | -4 |
|  |  |  | Paracingulate gyrus_L | 3.5 | -4 | 52 | -4 |
|  | DMN (PCC) | 290 | Parietal operculum cortex_R | 5.6 | 50 | -32 | 32 |
|  |  |  | Parietal operculum cortex_R | 5.3 | 52 | -32 | 28 |
|  |  |  | Supramarginal gyrus, anterior division_R | 5.3 | 60 | -28 | 30 |
|  |  |  | Supramarginal gyrus, posterior division_R | 3.1 | 56 | -36 | 34 |
|  |  | 274 | Superior parietal lobule_R | 4.5 | 38 | -48 | 56 |
|  |  |  | Angular gyrus_R | 4.1 | 42 | -50 | 52 |
|  |  |  | Lateral occipital cortex, superior division_R | 3.9 | 30 | -60 | 58 |
|  |  |  | Supramarginal gyrus, posterior division_R | 3.7 | 44 | -46 | 54 |
|  |  |  | Supramarginal gyrus, posterior division_R | 3.6 | 44 | -44 | 52 |
| YA | Left pHPC | 216 | Intracalcarine cortex_R | 5.1 | 10 | -74 | 14 |
|  |  |  | Intracalcarine cortex_R | 4.9 | 10 | -80 | 16 |
|  |  |  | Supracalcarine cortex_R | 4.7 | 4 | -76 | 16 |
|  |  |  | Cuneal cortex_R | 3.4 | 6 | -80 | 18 |
|  |  |  | Precuneus cortex | 3.4 | 12 | -62 | 16 |
| ^a^ Number of voxels  ^b^ Regions are labeled using the Harvard-Oxford Atlas  ^c^ Statistical threshold: voxel-level *p*-uncorrected < 0.005; cluster-level *p*-FDR corrected < 0.05  Abbreviations: DMN.PCC: Default mode network posterior cingulate cortex seed; L: Left hemisphere; Left pHPC: Left posterior hippocampus; OA: Older adults; R: Right hemisphere; YA: Young adults | | | | | | | |

# **Table S2. Resting-state functional connectivity patterns associated with sequence memory performance in older and young adults after covarying for age, sex and head motion**

| Group | Seed | Cluster size ^a^ | Brain region ^b^ | Peak statistics  (T) ^c^ | Peak MNI Coordinates | | |
| --- | --- | --- | --- | --- | --- | --- | --- |
|  |  |  |  |  | ***x*** | ***y*** | ***z*** |
| OA | Left pHPC | 373 | Lateral occipital cortex, superior division_L | 5.2 | -34 | -66 | 48 |
|  |  |  | Angular gyrus_L | 4.4 | -40 | -58 | 44 |
|  |  |  | Angular gyrus_L | 3.6 | -40 | -56 | 44 |
|  |  | 359 | Cingulate gyrus, posterior division | 4.9 | -12 | -42 | 42 |
|  |  |  | Precuneus cortex | 4.7 | -12 | -44 | 44 |
|  |  |  | Cingulate gyrus, posterior division | 4.5 | 0 | -34 | 34 |
|  |  | 189 | Frontal pole_R | 4.4 | 6 | 60 | -8 |
|  |  |  | Frontal pole_R | 3.9 | 0 | 58 | -8 |
|  |  |  | Frontal medial cortex | 3.6 | 0 | 52 | -8 |
|  |  |  | Paracingulate gyrus_R | 3.6 | 6 | 52 | -4 |
|  |  |  | Frontal pole_L | 3.6 | -4 | 60 | -8 |
|  |  |  | Paracingulate gyrus_L | 3.4 | -4 | 52 | -4 |
|  | DMN  (PCC) | 303 | Parietal operculum cortex_R | 5.5 | 50 | -32 | 32 |
|  |  |  | Supramarginal gyrus, anterior division_R | 5.2 | 60 | -26 | 30 |
|  |  |  | Parietal operculum cortex_R | 5.1 | 52 | -32 | 28 |
| YA | Left pHPC | 296 | Intracalcarine cortex_R | 5.1 | 10 | -82 | 16 |
|  |  |  | Occipital pole_R | 4.9 | 8 | -94 | 16 |
|  |  |  | Intracalcarine cortex_R | 4.9 | 12 | -72 | 12 |
|  |  |  | Supracalcarine cortex_R | 4.6 | 6 | -76 | 16 |
|  |  |  | Supracalcarine cortex_L | 3.6 | -2 | -82 | 14 |
|  |  |  | Cuneal cortex_R | 3.6 | 6 | -78 | 18 |
|  |  |  | Precuneus cortex | 3.4 | 10 | -60 | 12 |
| ^a^ Number of voxels  ^b^ Regions are labeled using the Harvard-Oxford Atlas  ^c^ Statistical threshold: voxel-level *p*-uncorrected < 0.005; cluster-level *p*-FDR corrected < 0.05  Abbreviations: DMN.PCC: Default mode network posterior cingulate cortex seed; L: Left hemisphere; Left pHPC: Left posterior hippocampus; OA: Older adults; R: Right hemisphere; YA: Young adults | | | | | | | |

# **3. Supplementary figures**

# **Figure S1. Between-group comparisons of resting-state functional connectivity in performance-relevant networks.**

**Fig. S1 Between-group comparisons of resting-state functional connectivity in performance-relevant networks.** Boxplots comparing functional connectivity strength between older adults and young adults for **(a)** left posterior hippocampus to default mode network clusters, **(b)** default mode network to posterior parietal cortex clusters, and **(c)** left posterior hippocampus to medial occipital cortex cluster. Box boundaries represent 25th and 75th percentiles, center lines indicate medians, and whiskers extend to minimum and maximum values. Individual data points are overlaid. Abbreviations: DMN: Default mode network; ICC: Intracalcarine cortex; OA: Older adults; pHPC: Posterior hippocampus; PPC: Posterior parietal cortex; YA: Young adults
